# Non-contact and nanometer-scale measurement of PN junction depth buried in Si wafers using terahertz emission spectroscopy

**DOI:** 10.1038/s41377-025-01911-0

**Published:** 2025-06-20

**Authors:** Fumikazu Murakami, Shinji Ueyama, Kenji Suzuki, Ingi Kim, Inkeun Baek, Sangwoo Bae, Dougyong Sung, Myungjun Lee, Sungyoon Ryu, Yusin Yang, Masayoshi Tonouchi

**Affiliations:** 1 Department of Electrical and Computer Engineering, Rice University, 6100 Main St., Houston, TX 77005 USA; 2 Advanced Equipment Lab, Samsung Device Solutions R&D Japan, 2-7 Sugasawa-cho, Yokohama Tsurumi-ku, Kanagawa 230-0027 Japan; 3 Core Technology R&D Team, Global Manufacturing & Infra Technology, Samsung Electronics Co. Ltd., 1-1 Samsungjeonja-ro, Hwaseong-si, Gyeonggi-do, 18448 Republic of Korea; 4 Advanced Process Development Team 4, Semiconductor R&D Center, Samsung Electronics Co. Ltd., 1-1 Samsungjeonja-ro, Hwaseong-si, Gyeonggi-do, 18448 Republic of Korea; 5 Metrology & Inspection Technology Team, Global Manufacturing & Infra Technology, Samsung Electronics Co. Ltd., 1-1 Samsungjeonja-ro, Hwaseong-si, Gyeonggi-do, 18448 Republic of Korea; 6 Process Development, Semiconductor R&D Center, Samsung Electronics Co. Ltd., 1-1 Samsungjeonja-ro, Hwaseong-si, Gyeonggi-do, 18448 Republic of Korea; 7 Institute of Laser Engineering, Osaka University, 2-6 Yamada-oka, Suita, Osaka, 565-0871 Japan; 8 Research Institute for Interdisciplinary Science, Okayama University, 3-1-1 Tsushimanaka, Kita-ku, Okayama, 700-8530 Japan

**Keywords:** Imaging and sensing, Terahertz optics, Ultrafast photonics

## Abstract

Buried channel array transistors enable fast and high-density integrated devices. The depth of the PN junction and carrier dynamics at the depletion layer in silicon wafers have a crucial influence on their performance and reliability. Therefore, rapid and non-contact/non-destructive inspection tools are necessary to accelerate the semiconductor industry. Despite the great efforts in this field, realizing a technique to probe the junction depth and carrier dynamics at the PN junction inside wafers remains challenging. Herein, we propose a new approach to access PN junctions embedded in wafers using terahertz (THz) emission spectroscopy. THz emission measurements and simulations demonstrate that the amplitude and polarity of THz emissions reflect the junction depth and carrier dynamics at the PN junctions. It allows us to evaluate the junction depth non-destructively with nanometer-scale accuracy, surpassing the limits of traditional techniques. Laser-induced THz emission spectroscopy is a promising method for the sensitive and non-contact/non-destructive evaluation of Si wafers and will benefit the modern semiconductor industry.

## Abstract (web summary)

THz emission from the PN junction inside wafers sensitively reflects the junction depth, enabling non-contact and nanometer-scale depth evaluation.

## Graphical abstract

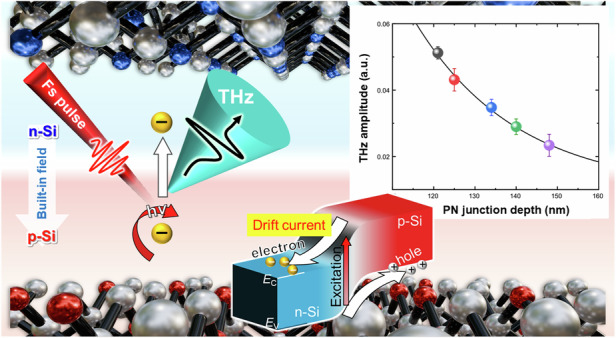

## Introduction

**Fig. 1:**
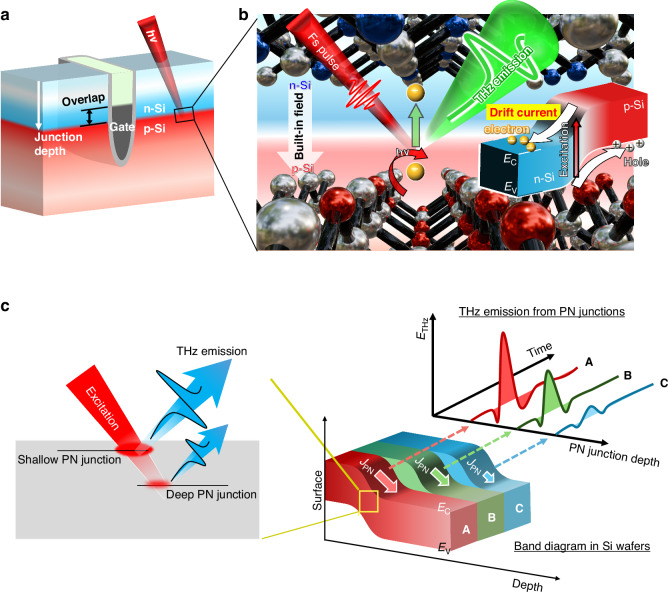
Concept of the non-contact inspection on the PN junction depth. **a** Schematic illustration of the buried channel transistor structure. **b** THz emission from the PN junction. Ultrafast photocarrier transport due to the built-in electric field (drift current) generates the THz electromagnetic waves at the PN junction. **c** PN junction depth dependence of the THz emission. A, B, and C represent the energy band diagram in Si wafers with different PN junction depths. Photocarrier density at PN junctions depends on their depth. Consequently, the amplitude of the THz emission from the PN junctions is sensitive to the depth

Silicon (Si)-based device technology is a key technology in modern society, and further miniaturization and three-dimensional integration of devices are essential to achieve high-density integration devices^1–4^. In particular, in the current explosive expansion of computing systems, the development of dynamic random access memory (DRAM) technology is significant because it determines the performance of central/graphics processing units (CPUs/GPUs). The access transistors in DRAMs require a low off-current and a high on-current simultaneously for quick and reliable operations^5^. Three-dimensional transistor structures show great potential for meeting these demands^5,6^. In particular, buried channel array transistors, such as saddle-fin transistors, in which gates are embedded within the wafer, have become mainstream for further enhancing integration^5,7^. To determine the device performance, controlling the quality of PN junctions is necessary. For example, the overlap length between the gate and n-doped layer affects the device characteristics such as the threshold voltage and drain-induced barrier lowering (Fig. 1a)^8,9^. And even a mere 10-nm change in the depth of the PN junction could affect the DRAM reliability^10^. In addition, the properties of the built-in electric field and the carrier dynamics within the depletion layer have important roles in developing high-performance devices^11,12^. Hence, evaluation techniques for them, especially non-destructive and non-contact methods, are indispensable.

Secondary ion mass spectrometry (SIMS) and spreading resistance profiling (SRP) are the most established tools for evaluating doping profiles^10,13^. These techniques allow for a very detailed analysis at the nanometer scale; however, they require destructive processes and a long measurement time for evaluation. To develop in-line evaluation tools, non-destructive/non-contact characterization methods have been intensively studied. Borden and Geiler demonstrated the junction depth evaluation using carrier illumination (CI) and photoluminescence heterodyne (PLH) techniques^14,15^. However, these techniques do not directly reflect the PN junction depth but the dopant concentration in highly doped layers, and their applicability to PN junction depth characterization remains unclear^14–16^. In addition, CI exhibits a complex response to graded profiles obtained by the ion implantation, which can lead to errors at the 20 nm level^17,18^. More recently, Raman and X-ray fluorescence (XRF) techniques have demonstrated non-destructive access to the doping profile^19,20^. Although non-destructive, non-contact approaches for doping density characterization are actively being studied, they remain challenging, and SIMS is the most commonly used method for detailed doping distribution analysis.

While characterization methods to access the doping density in wafers have been widely studied, non-destructive/non-contact techniques to approach the built-in field and carrier dynamics at PN junctions remain unavailable, and electrical characterization commonly relies on destructive and contact methods, such as SRP and 4-point probe measurements^10^. Musca and Hur demonstrated non-destructive approaches to the PN junction depletion field using laser beam induced current (LBIC) and electron beam induced current (EBIC); however, these techniques require electrode contacts^21,22^. Therefore, concrete methods to probe the carrier dynamics within the buried PN junction in a non-destructive and non-contact manner have not been established and are desired. In the present work, we propose the application of terahertz (THz) emission measurements for a non-destructive/non-contact evaluation.

THz emission spectroscopy (TES) and laser THz emission microscopy (LTEM) are emerging technologies that have been applied to advanced materials to phenomenologically discuss ultrafast photocarrier dynamics^23–29^, based on the concept of determining the built-in electric field and carrier dynamics in the materials^23,30^. On the other hand, THz radiation upon the femtosecond (fs) laser illumination involves various photocarrier scattering processes^31–33^, which requires complicated modeling for each individual case and hinders wide-ranging applications of TES and LTEM. To overcome the obstacle and expand the applicable research fields in semiconductor R&D, we recently proposed a simplified model that allows for the quantitative evaluation of various physical parameters of semiconductor materials and devices^34^, e.g., the surface potential of Si wafers^35^, the work function of VO_2_ films on Si wafers^36^, and various parameters of metal-insulator-semiconductor (MIS) structures^37–39^. These demonstrations have proven that TES/LTEM would be a useful analytical tool for monitoring and controlling semiconductor manufacturing processes and developing next-generation devices. On the other hand, while the THz emission from the semiconductor surface has been widely studied and described well by the emission models^34,35,37^, studies focusing on the THz emission from PN junctions remain limited. In particular, quantitative studies on the PN junctions by the THz emissions are still unexplored. To use TES in the field of semiconductor R&D, it is necessary to move TES up to a quantitative evaluation tool for PN junctions.

In the present study, we extend TES to investigate the PN junctions within wafers and demonstrate the evaluation of junction depth. We first propose a simplified model of the THz emission from PN junctions and demonstrate the correlation between the junction depth and THz emission amplitude. Subsequently, we perform the photocarrier dynamics simulations to analyze the optical response in the wafers and validate the TES results. Furthermore, we discuss the depth resolution of this technique and a potential approach for determining the junction depth using TES alone. These findings highlight TES as a promising tool for achieving rapid, non-contact, and sensitive characterization of Si wafers.

## Results

### Principle of the THz emission from the PN junction

We propose a simplified model for THz emission from PN junctions here. When photocarriers are excited within semiconductors, they are accelerated by the surface/interface built-in electric field, creating the transient drift current. The THz waves are generated by the drift current and defined by the time derivative of the photocurrent density, *J*_p_. In particular, when considering THz emission from a semiconductor surface, the peak amplitude of their electric field, ${E}_{{\rm{THz}}}$, is described as

1 $${E}_{{\rm{THz}}}\propto \frac{d{J}_{{\rm{p}}}}{{dt}}\propto \mu {E}_{{\rm{Max}}}{I}_{{\rm{p}}}$$

where *µ* is the carrier mobility, ${E}_{{\rm{Max}}}$ is the maximum electric field at the surface, and *I*_p_ is the excitation laser intensity^34^. In addition, when the penetration depth of the excitation light, ${\lambda }_{{\rm{L}}}$, is longer than the surface depletion layer width, *w*, ${I}_{{\rm{p}}}$ is transformed into the photocarrier density absorbed in the depletion region, $\frac{w}{{\lambda }_{{\rm{L}}}}{I}_{{\rm{p}}}$, and we obtain

2 $${E}_{{\rm{THz}}}\propto \pm \mu \frac{{V}_{{\rm{D}}}}{{\lambda }_{{\rm{L}}}}{I}_{{\rm{p}}}$$

where *V*_D_ is the diffusion potential^34^. As expressed by Eq. (2), the THz emission is primarily proportional to the carrier mobility and diffusion potential, which are defined by doping and defect densities, surface states, etc. Thus, TES can be applied to probe the material properties using this emission model, such as surface dipole, defects, and potential barriers in the superlattice structures^35,40,41^.

In contrast, the THz emission from PN junctions has been discussed phenomenologically based on the surface emission model^34^. Therefore, a simplified THz emission model for the PN junction is required to apply TES to the quantitative evaluation of the PN junction. When the excitation pulses illuminate the semiconductor, the laser intensity at the PN junction with a depth of *x*, *I*_p_(*x*), is expressed as

3 $${I}_{{\rm{p}}}\left(x\right)={I}_{{\rm{p}}0}\exp \left(-\frac{1}{{\lambda }_{{\rm{L}}}}x\right)$$

where *I*_p0_ denotes the laser intensity at the surface. Then, the surface electric field in Eq. (1) is replaced with the PN junction electric field, *E*_PN_. In addition, assuming that the depletion layer width at the PN junction is sufficiently small in highly doped wafers, the number of carriers contributing to THz radiation at the PN junction can be approximated by $w\,\times \,{I}_{{\rm{p}}0}\exp (-x/{\lambda }_{{\rm{L}}})$ (see Supplementary Fig. 1 for details about the equation). As a result, a simplified model describing the THz emission from the PN junction can be expressed as

4 $${E}_{{\rm{THz}}}\propto \mu {E}_{{\rm{PN}}}w{I}_{{\rm{p}}0}\exp \left(-\frac{1}{{\lambda }_{{\rm{L}}}}x\right)\propto \mu {V}_{{\rm{D}}}{I}_{{\rm{p}}0}\exp \left(-\frac{1}{{\lambda }_{{\rm{L}}}}x\right)$$

This suggests that the photocarrier density is larger at the PN junction with a shallower depth, generating the larger THz emission, which allowing for a non-destructive evaluation of junction depth as shown in Fig. 1. Note that the penetration depth of the typical fs laser at a wavelength of around 800 nm is much longer than the depth of PN junction in our samples^42^. Therefore, we employed 400 nm as an excitation wavelength in the present work (see Supplementary Fig. S2 for more details about choosing the wavelength).

### THz emission from the PN junction buried in the wafer

**Fig. 2:**
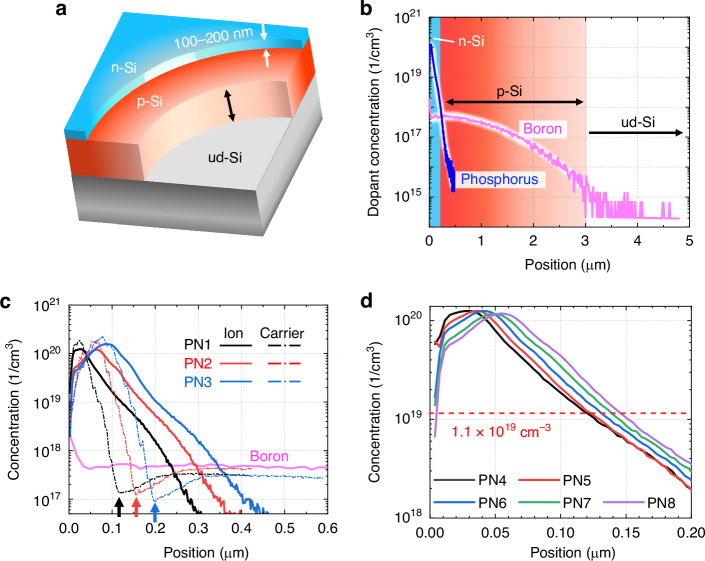
Schematic illustration of samples. **a** Structure of the Si wafer samples. The layers were formed in the following order: n-Si, p-Si, and ud-Si. **b** Dopant density distribution within the depth of 0–5 µm in the samples. The blue and pink lines represent the dopant concentrations of phosphorus and boron, respectively. The blue, red, and white layers correspond to the n-Si, p-Si, and ud-Si layers, respectively. **c** Detailed distribution of dopant and free-carrier concentrations in PN1–PN3. The black, red, and blue lines represent the distributions of PN1–PN3, respectively. The phosphorus ion concentrations are plotted as solid lines, and the carrier concentrations are plotted as dashed-dotted lines. The pink solid line indicates boron concentration. **d** Phosphorus concentrations in PN4–PN8 are depicted in black, red, blue, green, and purple lines, respectively

The structure and doping profiles of the samples used in this study are shown in Fig. 2. We prepared eight different wafers with different PN junction depths, named PN1–PN8. Each sample consisted of an n-Si layer with a 100–200 nm thickness, a p-Si layer with a thickness of approximately 3 µm, and an undoped Si (ud-Si) layer at the bottom. The PN junction depths in PN1–PN3 are 120, 160, and 200 nm (Fig. 2c), and PN4–PN8 have depths of 121, 125, 134, 140, and 148 nm (Fig. 2d), respectively. TES measurements were performed using an excitation wavelength of 400 nm with a laser power of 40 mW at an incidence angle of 45° on the sample surface. See the Method section for details about the samples and measurement setup.

**Fig. 3:**
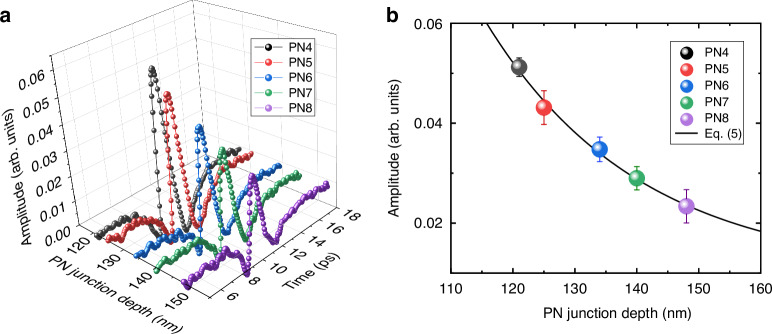
THz emission from the PN junctions. **a** PN junction depth dependence of the THz emission waveforms. The black, red, blue, green, and purple plots correspond to the emissions from PN4–PN8, respectively. **b** THz emission amplitude as a function of the PN junction depth. Each plot color corresponds to the waveforms in panel **a**. The black solid line represents the fitting curve using Eq. (5). Error bars represent the noise level

The proposed THz emission model expressed by Eq. (4) suggests that the THz emissions from wafers probe carrier dynamics at PN junctions and sensitively reflect the junction depth. To validate this model, we first measured THz emissions from wafers with various junction depths as shown in Fig. 3a. Although the differences in junction depth between samples are less than 10 nm, clear changes in the THz emission amplitudes were observed. The polarity of the waveforms represents the direction of the electric field where the THz waves were generated. Comparison with the emission from a reference sample confirmed that this polarity was consistent with the electric field at the PN junctions (see Supplementary Fig. S3). Therefore, the origin of the changes in the THz emissions was attributed to the PN junction depth, as described by Eq. (4).

### Junction depth dependence of the photocurrent and THz emission

To gain further insight into the properties of THz emission from the PN junctions, we conducted numerical simulations, compared them with experimental results, and explored carrier dynamics in the wafers. For measurements, we used wafers with PN junction depths of 120, 160, and 200 nm (PN1–PN3, respectively) and constructed corresponding wafer models, SIM1–SIM3, for simulations. Doping profiles in SIM1–SIM3 were defined based on those of PN1–PN3, respectively, as shown in Supplementary Fig. S4.

**Fig. 4:**
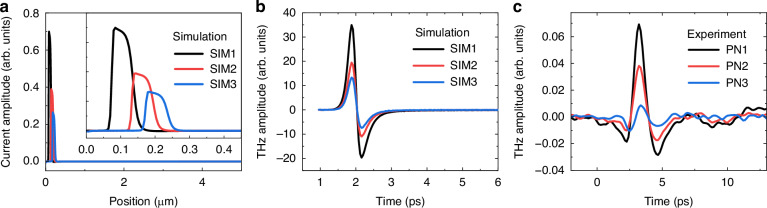
Carrier dynamics in the wafers and THz emission waveforms. **a** Photocurrent distributions in SIM1–SIM3. The inset shows a magnified view of 0–0.4 µm. **b** Calculated and **c** measured THz emission waveforms for SIM1–SIM3 and PN1–PN3, respectively. The black, red, and blue lines represent the plots for SIM1–SIM3 in panel **b** and for PN1–PN3 in panel **c**

Figure 4a shows the simulated photocurrent distributions in the wafers under 400 nm excitation. Positive and negative values on the vertical axis indicate the intensity of the photocurrents toward the interior and surface of the sample, respectively. Photocurrents were excited in the vicinity of PN junctions, and peak current intensity decreased with increasing PN junction depth, as shown in the inset. THz emissions from the wafers were estimated from these currents and plotted in Fig. 4b (see Supplementary Fig. S5 for the calculation). The larger the PN junction depth, the weaker the THz emission, which is consistent with the experimental observations for PN1–PN3, as shown in Fig. 4c. These results suggest that the THz emission amplitude from the wafers strongly reflects the PN junction depth. We also measured these samples using another THz technique, THz time-domain spectroscopy^43^, which shows the potential of TES (see Supplementary 6). On the other hand, THz emission waveforms with an excitation wavelength of 800 nm showed the opposite polarity of the electric field and less variation in their amplitudes (see Supplementary Fig. S7). This is attributed to the THz emission from the p-Si/ud-Si interface, indicating that the different excitation wavelengths can probe different depths and a precise excitation wavelength is required for the evaluation of the PN junction depth. For example, 500 nm or 632 nm excitation wavelengths can probe the PN junction according to their penetration depths^42^. However, because these lengths are much larger than the PN junction depth, the sensitivities in these wavelengths are expected to be worse than that in 400 nm excitation.

## Discussions

TES demonstrated that the amplitude and phase of the THz emissions sensitively reflect the depth and polarity of the built-in electric field at PN junctions, respectively. This suggests that TES probes the electric field and carrier dynamics at PN junctions inside wafers, which has not been achieved yet by other non-destructive methods. Furthermore, this technique can be used to map large-scale images^35,44^. Therefore, TES/LTEM meet the requirements for new Si wafer inspection tools: non-contact/non-destructive (no sample preparation), applicable to whole wafers, and nano-scale depth resolution.

In the present work, THz radiation electric fields can be approximated as shown in Eqs. (1) – (4). With this simplification, we consider the radiation comes from the vicinity of the junction boundary, which allows us to estimate the junction depth. As Yang and Sun reported, a mere variation in the PN junction depth of 10 nm affects device performance^9,10^. Their works were, however, based on transistors having a channel length of 20–30 nm. Further miniaturization will cause the short-channel effect, resulting in a more severe influence of the junction depth on the performance^45^. In addition, the precision of the position of peak doping concentration is considered to be ~10% of the target depth^46–48^. Therefore, the depth resolution of the evaluation method is important in determining its applicability and scope of application.

THz emission from the PN junction expressed by Eq. (4) describes only the THz emission from the PN junction inside the wafer; however, the THz emissions from Si wafers can include the surface emissions as well^35,37^. Furthermore, the generated THz waves are absorbed in the highly doped n-Si layer. Taking them into account, the detected THz emission amplitude as a function of the PN junction depth of *x* can be expressed as follows:

5 $${E}_{\mathrm{THz}}\left(x\right)={A}_{\mathrm{sur}}+B\cdot \exp \left(-\frac{1}{{\lambda }_{\mathrm{total}}}x\right)$$

6 $$\frac{1}{{\lambda }_{{\rm{total}}}}=\frac{1}{{\lambda }_{{\rm{L}}}}+\frac{1}{{\lambda }_{{\rm{THz}}}}$$

where *A*_sur_ is a component of the surface THz emission, *B* is a proportional constant, and ${\lambda }_{{\rm{THz}}}$ is the penetration depth for THz waves. We fitted the THz amplitudes in Fig. 3b using Eq. (5) and obtained *A*_sur_ = 0.007, *B* = 3.73, and ${\lambda }_{{\rm{total}}}=27\,{\rm{nm}}$. Strictly speaking, the n-Si layer has a depth-dependent doping density distribution and therefore exhibits different absorption at different depths^49^. In addition, since the doping profile varies in PN4–PN8, the penetration depth is expected to vary depending on the sample. However, the absorption is dominant in a high-doped region, and the peak concentration is almost the same across the samples. Therefore, we used ${\lambda }_{{\rm{total}}}=27\,{\rm{nm}}$ as a constant value across the samples in this section. In the determination of the depth resolution here, we calculated the minimum change in PN junction depth that produces a THz amplitude variation exceeding the noise level. It should be noted here that, although there can be several noise sources in measurements, the main noise source was attributed to the noise at a detector (see Supplementary 8 and Ref^41^.). To determine the noise level, we first calculated the standard deviation of the signal in the region before the THz peak appeared (4–8 ps in the time-domain waveforms shown in Fig. 3a). Subsequently, six times the standard deviation was defined as the peak-to-peak noise level. The error bars in Fig. 3b represent the calculated peak-to-peak noise level. By comparing this noise level with the changes in THz emission amplitude corresponding to variations in PN junction depth, calculated from Eq. (5), the depth resolution of the current system was estimated to be approximately 5 nm. In addition to the depth measurement, since the field distribution and carrier mobility are affected by doping concentrations as explained in Supplementary 1, we might be able to discuss them after many case studies. Note that TES is a complementary tool and conventional tools such as SIMS are required to extract some properties in wafers. However, once the calibration curves are established with the aid of existing tools, TES can enable the wafer scale in a rapid, non-contact, and non-destructive manner.

Furthermore, we discuss the incident angle dependence of the THz emission, with a special focus on depth analysis. When a sample surface is excited at an incident angle of *θ*_in_ the THz emission amplitude, *E*_THz_(*θ*_in_), normalized with respect to the THz emission amplitude at *θ*_in_ = 0 can be expressed as

7 $${\rm{Norm}}{E}_{{\rm{THz}}}\left({\theta }_{{\rm{in}}}\right)=\frac{{E}_{{\rm{THz}}}({\theta }_{{\rm{in}}})}{{E}_{{\rm{THz}}}(0)}=\exp \left(-\frac{1-\cos {\theta }_{{\rm{in}}}}{{\lambda }_{{\rm{L}}}{\cos \theta }_{{\rm{in}}}}x\right)$$

As shown in this equation, the effect of variation in material parameters can be subtracted. Therefore, this approach has the potential to enable quantitative evaluation without relying on additional methods or reference samples (see Supplementary 9), which makes TES a more powerful tool. When this method is applied to actual measurements, however, the phased-array effect can affect the far-field detection^50,51^. More detailed studies on the phased-array effect on the THz emission are needed to solve this issue. In addition, near-field detection systems, such as Teraspike, could offer another solution.

The evaluated resolution calculated by Eq. (5) is better than the value reported by Yang and Sun^9,10^. However, further downsizing devices requires a better resolution due to the short-channel effect^45^. Here, excitation with a higher incident angle can make the technique more sensitive to the junction depth because the large incident angle shortens the effective penetration length along the depth direction (${\lambda }_{{\rm{eff}}}({\theta }_{{\rm{in}}})={\lambda }_{{\rm{L}}}\cos {\theta }_{{\rm{in}}}$, where ${\lambda }_{{\rm{eff}}}$(*θ*_in_) is the effective penetration depth at an incident angle of ${\theta }_{{\rm{in}}}$). In addition, the sensitivity of THz detection presents another limitation to the depth resolution. The noise level of the current detection system is comparable to the variation in THz emission amplitude caused by a ~5 nm change in PN junction depth. Therefore, higher detection sensitivity, ultimately at the level of single-photon detection, is required to achieve ultra-high-resolution PN junction depth evaluation. Recently, parametric frequency upconversion, an ultra-sensitive detection method, and THz photomultiplier tubes (THz-PMT) have been actively studied^52,53^. Therefore, further improvement in the depth resolution can be achieved by combining the high incident angle with the sensitive detection methods. Furthermore, the main focus of this work is the study of the THz emission from PN junctions and the demonstration of the evaluation of the relative PN junction depth by THz emissions. Therefore, at this stage, this work did not use a reference for calibration to estimate the actual depth value. To determine the exact depth from a single THz waveform, a reference wafer with a known junction depth is required. Furthermore, the influence of the doping parameters on the THz emission, such as peak doping concentration, ion flux, acceleration voltage, and annealing temperature, should be revealed to draw a calibration curve to find the absolute depth of the PN junction from a single THz waveform.

As a final note, measurements in the present work were performed using a 45-degree incident angle with a defocused laser spot. This configuration was employed to enhance the THz emission by the phased-array effect^50,51^. Also, the quasi-reflection detection angle was used to enhance the sensitivity to PN junction depths (see Supplementary Fig. 10). This setup can improve the signal-to-noise ratio, however, the excitation beam size limits the 2D spatial resolution in LTEM mappings. In addition, a higher incident angle can obtain a better depth resolution as mentioned in the discussion of the depth resolution, however, a large incident angle broadens the illumination spot size. Therefore, regarding the incident angle and beam spot size, the relationship between the depth resolution and 2D spatial resolution is a trade-off. On the other hand, there is no upper limit to the wafer size for the LTEM scan^35,44^.

In conclusion, we demonstrated the non-destructive and non-contact evaluation of the PN junction depth using THz emission spectroscopy. Experimental measurements and numerical simulations showed that the amplitude and phase of the THz radiation sensitively reflect the depth and polarity of the built-in electric field at the PN junction. This suggests that TES can evaluate the built-in field at the PN junction buried inside wafers and carrier dynamics within the depletion field, which has not been realized using existing approaches. We estimated the depth resolution of the current system to be ~5 nm, calculated using the simplified THz emission model. These results suggest that TES/LTEM paves the way for the realization of a non-contact and non-destructive inspection tool for PN junctions buried inside wafers, surpassing the limitations of current approaches. This technique will enable us to find electrical defects in the early stages of the device process and, consequently, to approach problems easily during subsequent events in the process. As a result, TES will help to reduce a large amount of water and resources consumed in the manufacturing process, as well as the cost of later-stage or post-production inspection and disposal of defective products. TES will be an attractive and important technology for both developing highly advanced devices and moving toward a sustainable society.

## Materials and methods

### Samples

Eight samples with different doping profiles were prepared. Each sample consisted of an n-Si layer (doped with phosphorus, 100–200 nm), a p-Si layer (doped with boron, approximately 3 µm), and an ud-Si layer at the bottom, as shown in Fig. 2a, b, and the total wafer thickness was $675\pm 25$ µm. The boron-doping profiles were consistent across all the samples. Figure 2c shows the profiles of the dopant and free-carrier densities for PN1–PN3. The dopant and carrier densities were measured using SIMS and SRP, respectively. The PN junctions were estimated to be formed at depths of 120, 160, and 200 nm, where the carrier density was minimized owing to the compensation effects (black, red, and blue arrows). The other samples, PN4–PN8, were designed to have differences in PN junction depth of less than 10 nm, as shown in Fig. 2d. We estimated the PN junction depths to be 121, 125, 134, 140, and 148 nm, respectively, where the phosphorus density was $1.1\times {10}^{19}\,{\mathrm{cm}}^{-3}$. This value corresponds to the phosphorus concentration at the PN junctions in PN1–PN3.

### Terahertz emission spectroscopy

**Fig. 5:**
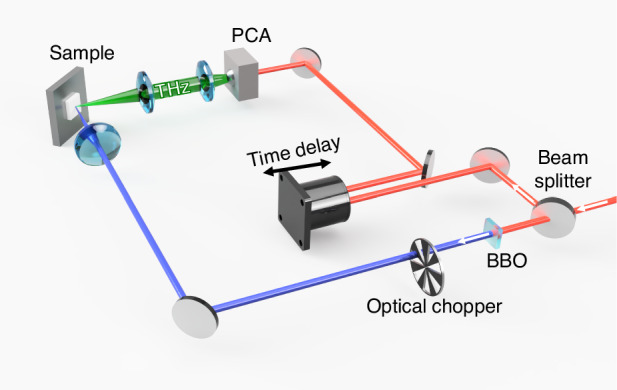
Schematic illustration of the TES measurement system

Figure 5 shows a schematic diagram of the TES system used in this study. Fs pulses (center wavelength: 800 nm; pulse width: 120 fs; repetition frequency: 80 MHz) obtained from a Ti:sapphire laser (Spectra Physics, MaiTai) were used as the excitation source. The pulses were split into probe pulses for detector operation and pump pulses for sample excitation using a beam splitter. The pump pulses were converted into a 400 nm wavelength through a beta-barium borate (BBO) crystal and focused onto the n-layer side of the samples at an incident angle of 45° with a diameter of approximately 2 mm. The THz emission at the quasi-reflection angle was focused onto a photoconductive antenna (PCA) detector using two THz lenses. We used a comb-shaped PCA (Thorlabs, PCA800) as a detector. At the PCA, photocarriers are excited by the probe pulses and driven by the incident THz pulses, generating a transient photocurrent. This current is proportional to the magnitude of the THz electric field. A time-delay stage controls the approach timing of probe pulses to monitor the THz electric field at different time positions, which allows for the measurement of time-resolved THz waveforms. The detailed principle of the detection can be found elsewhere^54^.

### Carrier dynamics simulation

We conducted carrier dynamics simulations on Si wafers using COMSOL MULTIPHYSICS software. The analytical solution was obtained by solving the combined drift-diffusion and Poisson’s equations. In real measurements, transient currents flow in the direction normal to the wafer surface owing to carrier acceleration in the depletion region of the PN junction. Therefore, we constructed a one-dimensional model in the depth direction for the simulation. The dopant distributions in SIM1–SIM3 were defined according to those in PN1–PN3 shown in Fig. 2c (see Supplementary Fig. S4 for the doping profiles in the COMSOL simulation models).

When the excitation light was incident on a semiconductor surface, the intensity of the light at a depth of *x* is represented by Eq. (3). Assuming that the decrease in intensity is due to photocarrier generation by single-photon absorption, the distribution of the photoexcited carrier density in the Si wafers, *N*_exc_(*x*), is expressed as follows:

8 $${N}_{\mathrm{exc}}\left(x\right)=\frac{1}{{\lambda }_{{\rm{L}}}}{I}_{{\rm{p}}0}\exp \left(-\frac{1}{{\lambda }_{{\rm{L}}}}x\right)$$

We employed the penetration depths of 0.1 and 10 µm for the excitation wavelengths of 400 and 800 nm, respectively^42^. The mobility and lifetime of carriers in the wafers, $\mu \left({N}_{{\rm{i}}}\right)$ and $\tau \left({N}_{{\rm{i}}}\right)$, are expressed as follows:

9 $$\tau {\left({N}_{{\rm{i}}}\right)}^{-1}={\tau }_{{\rm{R}}}^{-1}+{C}_{{\rm{p}}}{N}_{{\rm{i}}}^{2}$$

10 $$\mu \left({N}_{{\rm{i}}}\right)={\mu }_{\min }+\frac{{\mu }_{\max }-{\mu }_{\min }}{1+{\left({N}_{{\rm{i}}}/{N}_{{\rm{ref}}}\right)}^{\alpha }}$$

where ${\tau }_{{\rm{R}}}$ is the recombination lifetime and *C*_p_ is the Auger coefficient^55,56^. The values of *μ*_min_, *μ*_max_, *N*_ref_, and $\alpha$ were referenced from Ref.^56^. These values are listed in Table 1.

**Table 1:** Values of parameters in Eqs. (9) and (10)^55,56^

Parameter	Electron	Hole	Units
${\tau }_{{\rm{R}}}$	40	40	µs
*C*_p_	10^−31^	10^−31^	cm^6^ s^−1^
*μ*_min_	92	47.7	cm^2^ V^−1^ s^−1^
*μ*_max_	1360	495	cm^2^ V^−1^ s^−1^
*N*_ref_	1.3 × 10^17^	6.3 × 10^16^	cm^−3^
*α*	0.91	0.76	–

## Supplementary information

Supplementary Information for Non-contact and nanometer-scale measurement of PN junction depth buried in Si wafers using terahertz emission spectroscopy.

## Data Availability

The data that support the findings of this study are openly available in figshare at 10.6084/m9.figshare.28766750.
