# Supplementary material for: Non-contact and nanometer-scale measurement of PN junction depth buried in Si wafers using terahertz emission spectroscopy
Source: Light Sci Appl. 2025 Jun 20;14:216. doi: 10.1038/s41377-025-01911-0 (PMC12179313; doi:10.1038/s41377-025-01911-0)
Supplement: Supplementary file 1 — Supplementary Information for Non-contact and nanometer-scale measurement of PN junction depth buried in Si wafers using terahertz emission spectroscopy. [file 41377_2025_1911_MOESM1_ESM.docx]

Supplementary Information for

**Non-contact and nanometer-scale measurement of**

**PN junction depth buried in Si wafers**

**using terahertz emission spectroscopy**

Fumikazu Murakami^1^, Shinji Ueyama^2^, Kenji Suzuki^2^, Ingi Kim^3^, Inkeun Baek^4^, Sangwoo Bae^3^, Dougyong Sung^3^, Myungjun Lee^4^, Sungyoon Ryu^5^, Yusin Yang^6^,Masayoshi Tonouchi^7,8,*^

Affiliations

*^1^Department of Electrical and Computer Engineering, Rice University, 6100 Main St., Houston, TX, 77005, USA*

*^2^Advanced Equipment Lab, Samsung Device Solutions R&D Japan, 2-7 Sugasawa-cho, Yokohama Tsurumi-ku, Kanagawa 230-0027, Japan*

*^3^Core Technology R&D Team, Global Manufacturing & Infra Technology, Samsung Electronics Co. Ltd., 1-1 Samsungjeonja-ro, Hwaseong-si, Gyeonggi-do, 18448, Republic of Korea*

*^4^Advanced Process Development Team 4, Semiconductor R&D Center, Samsung Electronics Co. Ltd., 1-1 Samsungjeonja-ro, Hwaseong-si, Gyeonggi-do, 18448, Republic of Korea*

*^5^Metrology & Inspection Technology Team, Global Manufacturing & Infra Technology, Samsung Electronics Co. Ltd., 1-1 Samsungjeonja-ro, Hwaseong-si, Gyeonggi-do, 18448, Republic of Korea*

*^6^Process Development, Semiconductor R&D Center, Samsung Electronics Co. Ltd., 1-1 Samsungjeonja-ro, Hwaseong-si, Gyeonggi-do, 18448, Republic of Korea*

*^7^Institute of Laser Engineering, Osaka University, 2-6 Yamada-oka, Suita, Osaka 565-0871, Japan*

*^8^Research Institute for Interdisciplinary Science, Okayama University, 3-1-1 Tsushimanaka, Kita-ku, Okayama 700-8530, Japan*

^*^Author to whom correspondence should be addressed

Email: tonouchi@okayama-u.ac.jp

**Supplementary 1: Simplified model describing the THz emission from the PN junction.** In this section, we discuss the THz emissions from the PN junction. We assumed here that the doping profile has a linearly graded profile in the depletion region of the PN junction and expressed the slope of the linear distribution as *a* and *b* in the n- and p-doped regions, respectively. Supplementary Fig. S1 shows the dopant profile near the PN junction, and$N_{d\_eff}=ax_{n}$ and $N_{a\_eff}=bx_{p}$ represent effective doping concentrations at the edge of the PN junction ($x=x_{n}, x_{p}$ are depths of the edge of the PN junction), respectively. According to the Poisson equation, the potentials on the n- and the p-layer side, $\psi_{n}(x)$ and $\psi_{p}(x)$ are expressed as

| $\frac{\partial^{2}\psi_{n}\left( x \right)}{\partial x^{2}}=-\frac{eax}{\varepsilon_{\mathrm{Si}}\varepsilon_{0}}, \left( 0<x<x_{n} \right)$ | (S1) |
| --- | --- |
| $\frac{\partial^{2}\psi_{p}\left( x \right)}{\partial x^{2}}=-\frac{ebx}{\varepsilon_{\mathrm{Si}}\varepsilon_{0}}, \left( x_{p}<x<0 \right)$ | (S2) |

where *e* is the elementary charge, $\varepsilon_{\mathrm{Si}}$ is the dielectric constant of silicon, and $\varepsilon_{0}$ is the vacuum permittivity. The boundary conditions are given by

| $\frac{\partial\psi_{p}\left( x_{p} \right)}{\partial x}=0, \frac{\partial\psi_{n}\left( x_{n} \right)}{\partial x}=0$ | (S3) |
| --- | --- |
| $\psi_{p}\left( x_{p} \right)=0, \psi_{n}\left( x_{n} \right)=V_{D}$ | (S4) |
| $\frac{\partial\psi_{p}\left( 0 \right)}{\partial x}=\frac{\partial\psi_{n}\left( 0 \right)}{\partial x}$ | (S5) |
| $\psi_{p}\left( 0 \right)=\psi_{n}\left( 0 \right)$ | (S6) |

where *V*_D_ represents the diffusion potential. From Eqs. (S1) – (S4), we obtain

| $\psi_{n}\left( x \right)=-\frac{ea}{{6\varepsilon}_{\mathrm{Si}}\varepsilon_{0}}\left( x^{3}-3x_{n}^{2}x+2x_{n}^{3} \right)+V_{D}$ | (S7) |
| --- | --- |
| $\psi_{p}\left( x \right)=-\frac{eb}{{6\varepsilon}_{\mathrm{Si}}\varepsilon_{0}}\left( x^{3}-3x_{p}^{2}x+2x_{p}^{3} \right)$ | (S8) |

In addition, we obtain the following from Eqs. (S5) – (S8):

| $V_{D}=\frac{e}{3\varepsilon_{\mathrm{Si}}\varepsilon_{0}}\left( ax_{n}^{3}-bx_{p}^{3} \right)$ | (S9) |
| --- | --- |
| $ax_{n}^{2}=bx_{p}^{2}$ | (S10) |

Therefore, the positions at the edge of the PN junction and the depletion layer width are given by

| $x_{n}=\frac{3\varepsilon_{\mathrm{Si}}\varepsilon_{0}}{eN_{d\_eff}(x_{n}-x_{p})}$ | (S11) |
| --- | --- |
| $x_{p}=-\frac{3\varepsilon_{\mathrm{Si}}\varepsilon_{0}}{eN_{a\_eff}(x_{n}-x_{p})}$ | (S12) |
| $w=x_{n}-x_{p}=\sqrt{\frac{3\varepsilon_{\mathrm{Si}}\varepsilon_{0}V_{D}}{e}\frac{N_{d\_eff}+N_{a\_eff}}{N_{d\_eff}N_{a\_eff}}}$ | (S13) |

Since the THz emission is mainly generated in the region with a strong built-in electric field in the PN junction, *E*_B_ in Eq. (1) in the main text can be replaced by $E_{\max}={\partial\psi(0)}/{\partial x}$. In addition, the number of carriers contributing to the THz radiation at a depth *x* can be described by $w\times I_{p0}\exp({-x}/{\lambda_{L}})$ by assuming that the depletion layer width is sufficiently small and the absorption of photons is uniform within the depletion layer width. As a result, a simplified model describing the THz emission from the PN junction can be expressed as

| $E_{\mathrm{THz}}\propto\mu E_{\max}wI_{p0}\exp\left( -\frac{1}{\lambda_{L}}x \right)\propto\mu V_{D}I_{p0}\exp\left( -\frac{1}{\lambda_{L}}x \right)$ | (S14) |
| --- | --- |

where $\mu$ is the carrier mobility, $I_{p0}$ is the illumination photon density at the surface, and $\lambda_{L}$ is the penetration depth of an excitation pulse. This formula indicates that the THz emission amplitude depends on the PN junction depth. This is the basis of the concept of the PN junction depth evaluation using TES. Furthermore, considering the diffusion potential $V_{D}=\left( {k_{B}T}/e \right)\ln\left( {N_{d\_eff}N_{a\_eff}}/{n_{i}^{2}} \right)$, where *k*_B_ is the Boltzmann constant, *T* is temperature, and *n*_i_ is the intrinsic carrier density in Si, the dopant distribution and dopant density near the PN junction can be reflected in the THz emissions. However, the dopants in PN4–PN8 have similar distributions near the PN junction as shown in Fig. 2, which indicates that the differences in $N_{d\_eff}$ and $N_{a\_eff}$ between the samples are considered negligible within the scope of this study. Therefore, in the present work, we focused on evaluating the PN junction depth at the nanometer scale.


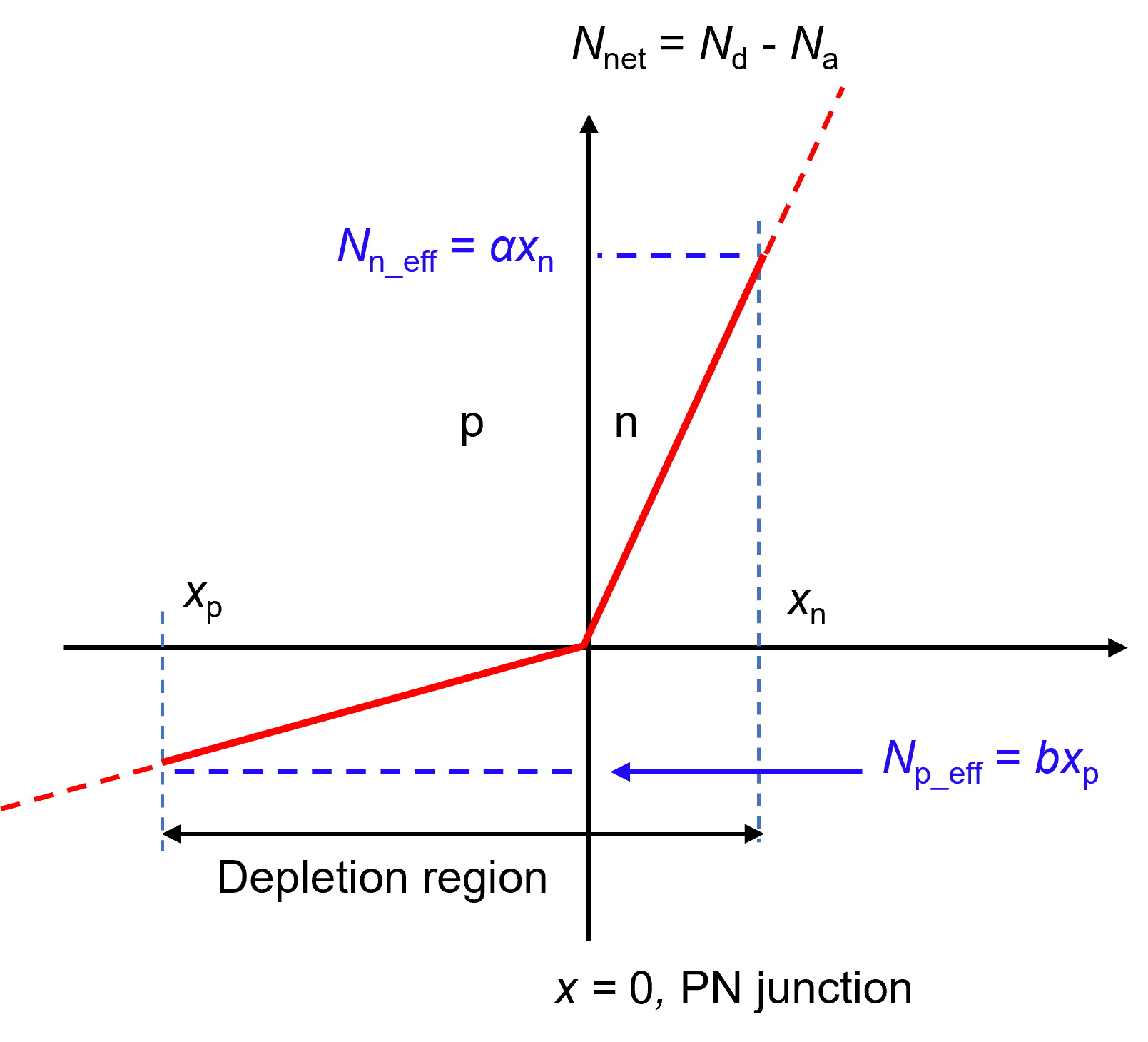


**Supplementary Fig. S1 | Configuration of the doping distribution near the PN junction.**

**Supplementary 2: Relationship between an excitation wavelength and measurement of PN junction depth.** Before the experiments, we first calculated the THz emission electric field radiated from the PN junction to find the most suitable wavelength for measurements, because the penetration depth of the excitation laser varies with different wavelengths. When the target depth of the PN junction is *x* nm and the PN junction depth in the actual wafer is *x+*Δ*d* (Δ*d* is the error in the PN junction depth), ${\Delta E}_{\mathrm{THz}}\left( x \right)=E_{\mathrm{THz}}\left( x \right)-E_{\mathrm{THz}}\left( x+\Delta d \right)$ represents the difference in the THz emission amplitudes from the PN junctions at depths of *x* and *x+*Δ*d*. Considering the case where the depth error Δ*d* is minimized to the limit, the difference between these two THz-wave radiation amplitudes is expressed as

| ${\Delta E}_{\mathrm{THz}}\left( x \right)=E_{\mathrm{THz}}\left( x \right)-E_{\mathrm{THz}}\left( x+\Delta d \right)\propto\frac{\partial E_{\mathrm{THz}}\left( x \right)}{\partial x}$ | (S15) |
| --- | --- |

*E*_THz_(*x*) is defined by Eq. (4). When $\Delta E_{\mathrm{THz}}(x)$ is larger than the noise value, the error in junction depth is detected. Therefore, a wavelength that has the biggest $\Delta E_{\mathrm{THz}}(x)$ value is the most favorable in the present work. We calculated $\Delta E_{\mathrm{THz}}(x)$ values at different excitation wavelengths, as shown in Supplementary Fig. S2. PN4–PN8 (samples for measurements) have the PN junction depth of 121–148 nm, and an excitation wavelength of 400 nm is the most favorable for *x* = 130 nm. Thus, this wavelength is employed in the present work.

**
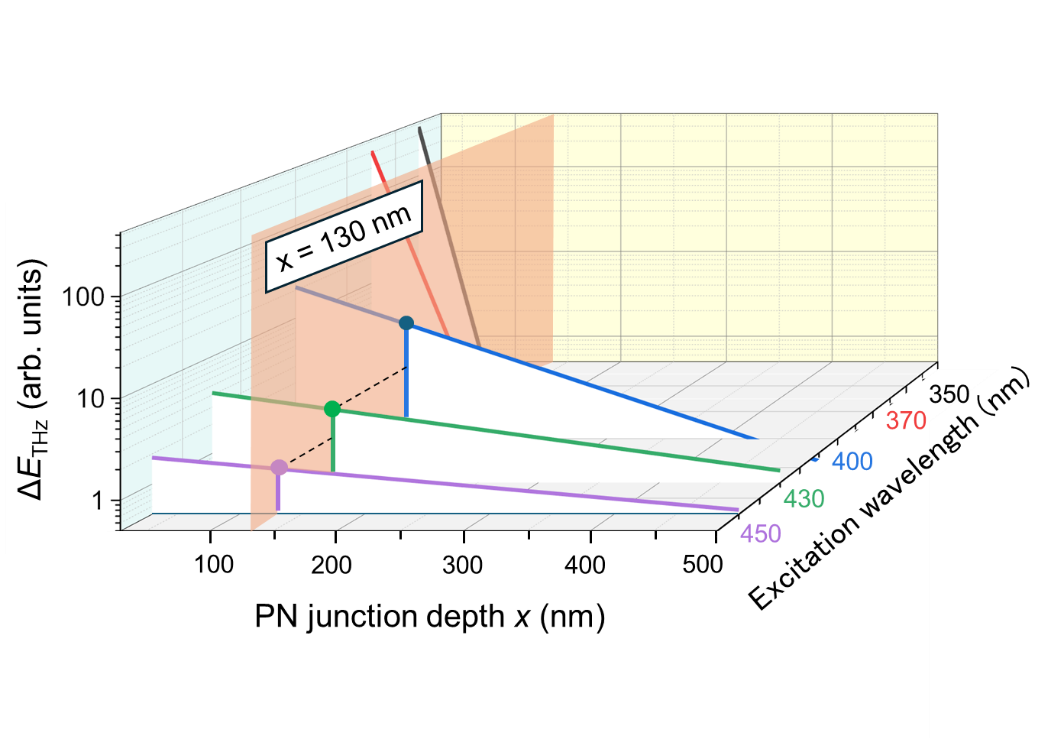
**

**Supplementary Fig. S2 | Relationship between the excitation wavelength and THz emission from PN junctions.** Black, red, blue, green, and purple lines correspond to the $\Delta E_{\mathrm{THz}}(x)$ value at 350, 370, 400, 430, and 450 nm, respectively. The values on the orange plane represent $\Delta E_{\mathrm{THz}}(x)$ values at *x* = 130 nm.

**Supplementary 3: Direction of the transient current inside Si wafer samples.** To estimate the current direction, we compared the THz emission from the Si wafer sample (PN1) with that from InAs, as shown in Supplementary Fig. S3. These THz emissions showed a polarity inversion (main peaks are depicted with red and black arrows for PN1 and InAs, respectively), which means that the photoexcited carriers in these two samples move in opposite directions. Given that the transient photocurrent in InAs flows towards the sample surface owing to the photo-Dember effect,^1,2^ this result indicates the photocurrent in the Si wafer samples towards the interior of the sample, which agrees with the electric field of the PN junction.

**
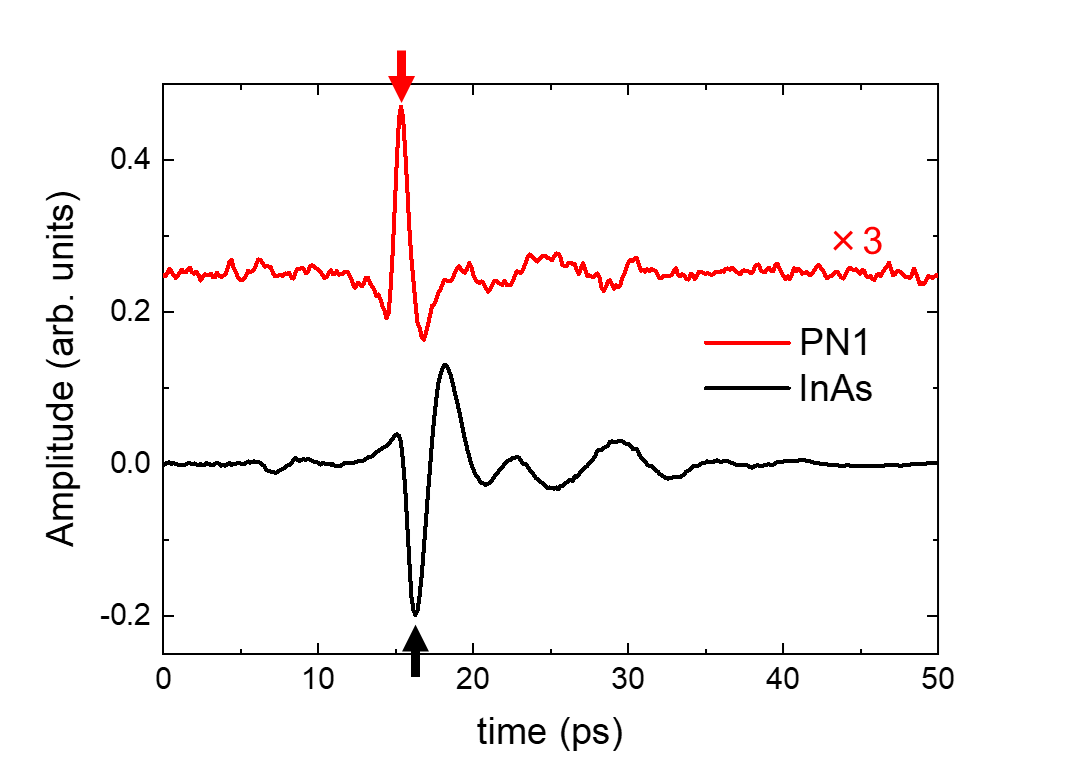
**

**Supplementary Fig. S3 | THz emissions from Si wafer sample and InAs.** The red and black lines represent the THz waveforms emitted from PN1 and InAs, respectively. The red and black arrows represent the main peak for each THz waveform.

**Supplementary 4: Simulation models.** Supplementary Figures S4a–S4c show the distributions of free-carrier and acceptor densities in the real samples (PN1–PN3). Note that we used the free-carrier distribution to determine the activated donor distribution because the SIMS result represents both activated and inactivated donor ions. Supplementary Figures S4d–S4f show the donor and acceptor distributions in the samples in simulations (SIM1–SIM3).


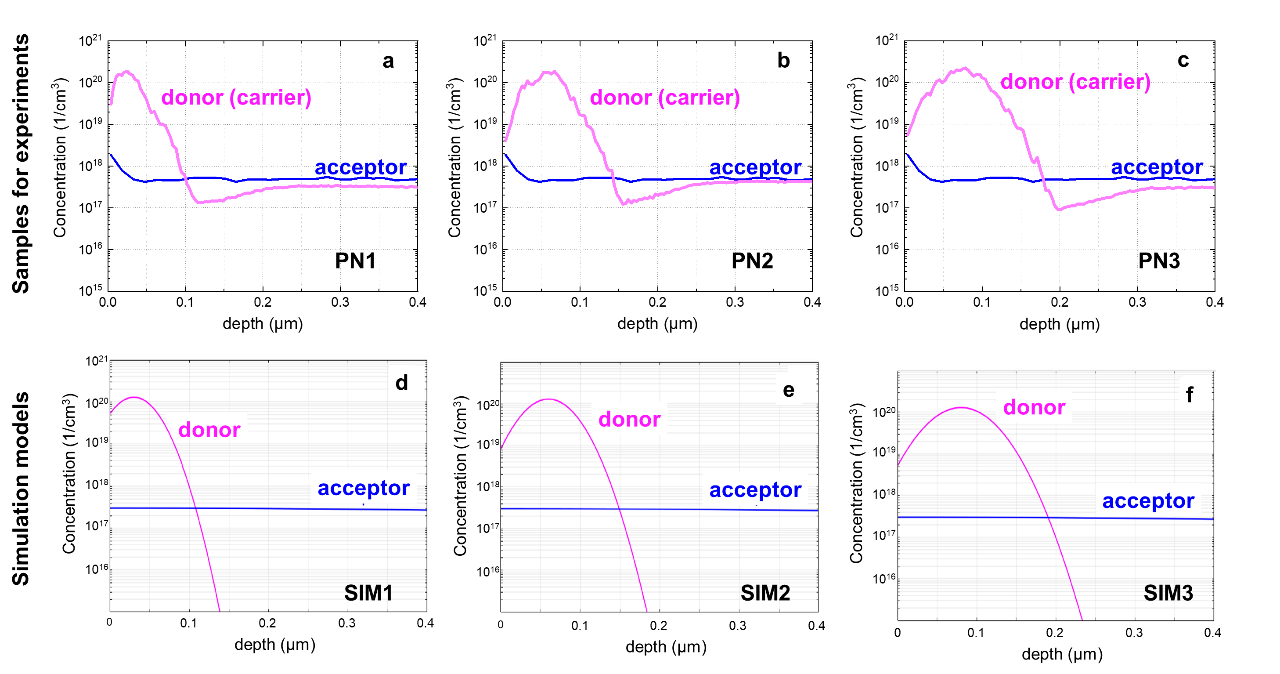


**Supplementary Fig. S4 | Donor and acceptor distributions in the simulation models.** The pink and blue lines represent the donor and acceptor distributions, respectively. **a–c** Experimentally measured carrier density (pink lines) and boron distribution (blue lines) in PN1–PN3. **d–f** Numerically determined donor (pink lines) and acceptor (blue lines) densities in SIM1–SIM3.

**Supplementary 5: Simulations of transient photocurrent and THz emission.** In the carrier dynamics simulation, the spatial distributions of photocurrent under 400 nm excitation were calculated at intervals of 0.04 ps for SIM1–SIM3. Supplementary Figure S5a shows the photocurrent distribution in SIM1 at some point between 1.2 and 5.44 ps. The current amplitude increased from 1.2 to 2 ps and decreased after 2 ps. The current distributions in SIM1–SIM3 at 2 ps are plotted in Fig. 4a. The THz emissions are expressed by the time derivation of the transient current as shown in Eq. (1). We obtained the time variation of the currents plotted in Supplementary Fig. S5b by integrating the current intensity across the entire region in the simulation models at each time point and calculated the THz emission waveforms, as shown in Fig. 4b.

**
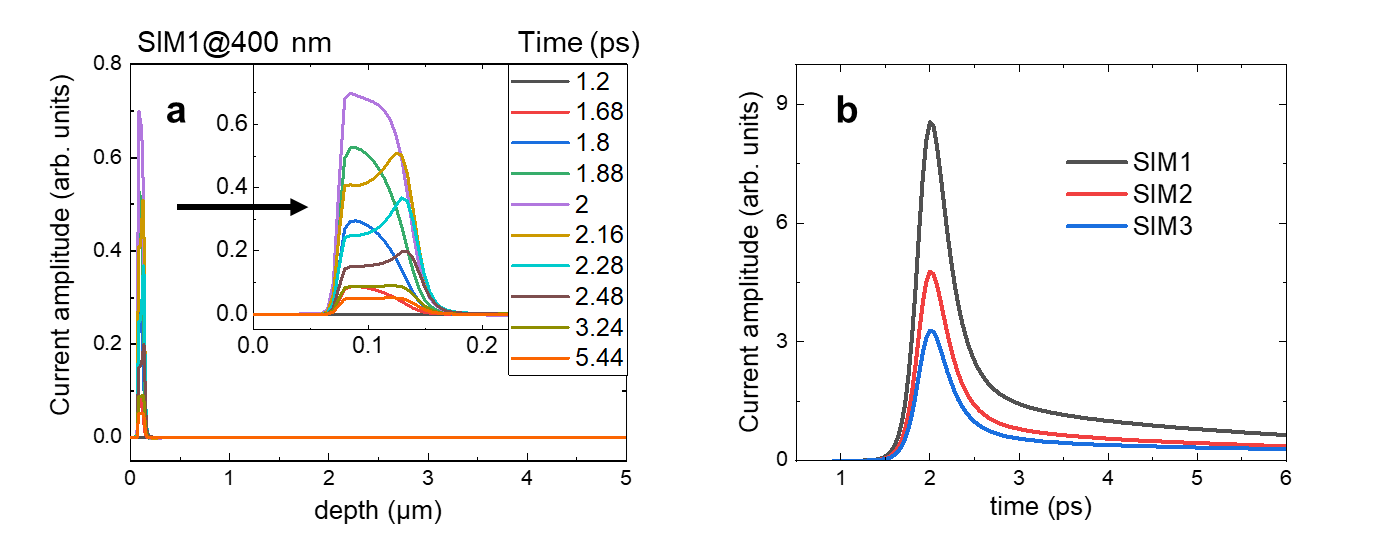
**

**Supplementary Fig. S5 | Transient current simulation under 400 nm excitation. a** The photocurrent distribution in SIM1 was simulated for 0–6 ps at intervals of 0.04 ps, and some of them are displayed. **b** Time variation of the currents in the wafers. The black, red, and blue lines represent the transient currents for SIM1–SIM3, respectively.

**Supplementary 6: THz-TDS on Si wafers.** Supplementary Figure S6 shows the result of THz time-domain spectroscopy (THz-TDS) on PN1 – PN3. A black line represents the reference (incident) THz pulse, and the other lines show the THz waveforms transmitted through PN1–PN3. The information obtained by TES and THz-TDS for Si wafer differs in the following points: TES observes the response of photocarriers, such as the transient acceleration of carriers, injected into the depletion layer of the PN junction. As a result, it reveals the polarity and intensity of the surface/interface electric fields. On the other hand, THz-TDS probes the interaction between the material and THz waves, such as the refractive index and THz absorption. The THz absorption within the n-Si layer can reflect the n-Si layer thickness (= PN junction depth). However, the sensitivity to the junction depth shown in Supplementary Fig. S6 is much smaller than that in TES, as shown in Fig. 4. This indicates that TES is necessary to characterize the PN junction depth formed inside a wafer.


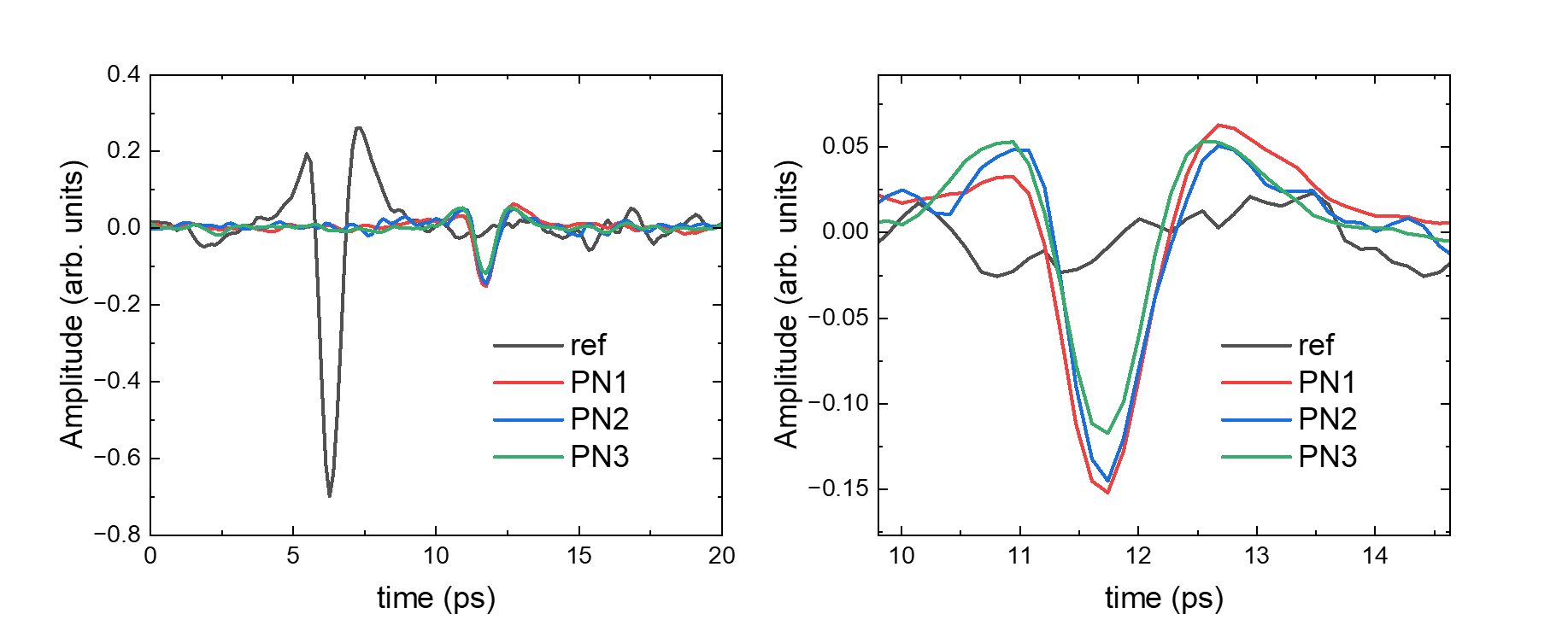


**Supplementary Fig. S6 | THz-TDS measurement on PN1 – PN3.** The black line represents the illuminated THz pulse. Red, blue, and green lines represent the THz waveforms transmitted through PN1, PN2, and PN3, respectively.

**Supplementary 7: THz emission from p-Si/ud-Si interface.** In the result section in the main text, we focused on the THz emission from the PN junction with an excitation wavelength of 400 nm; however, the wafers have another interface that could emit the THz waves: the p-Si/ud-Si interface. To discuss the influence of this interface on the THz emission, we obtained the THz emission under 800 nm excitation and compared it with that under 400 nm excitation. The measured and calculated THz emission waveforms for PN1 and SIM1 are plotted in Supplementary Figs. S7a and S7b, respectively. In both the experiment and simulation, the THz electric fields excited by the two different wavelengths show polarity flipping (blue and red arrows). As the polarity of THz waves reflects the direction of the built-in electric field in materials, the polarity flipping means that the THz emission under 800 nm excitation was generated at an area different from the PN junction. The carrier dynamics simulations for both wavelengths are depicted in Supplementary Fig. S7c. This simulation revealed that the photocurrent under 800 nm excitation was generated at a depth of approximately 3 µm as well, which is in the vicinity of the junction between p-Si and ud-Si, while no current in this area was observed under 400 nm excitation. This current has a polarity opposite to the current at the PN junction, resulting in the polarity flipping in the THz waves. In addition, the THz emission waveforms and current distribution for PN3 and SIM3 excited at a wavelength of 800 nm are plotted as pink dash-dotted lines in Supplementary Fig. S7. While change in the depth of the PN junctions is reflected in the position of the current (illustrated with a white arrow in Supplementary Fig. S7c), no significant change in the THz waveforms was observed in either the experiment or simulation. This indicates that the THz emissions at an excitation wavelength of 800 nm reflect the electric field at the p-Si/ud-Si junction rather than the electric field at the PN junction. Therefore, a precise excitation wavelength should be selected for the target depth of the PN junctions.


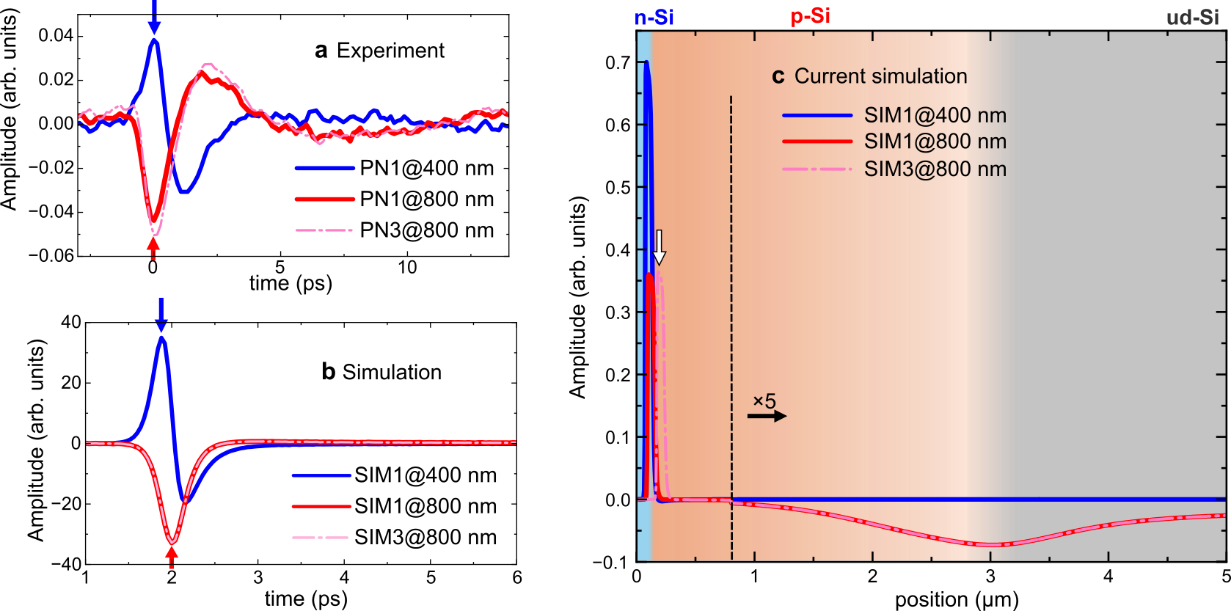


**Supplementary Fig. S7 | THz emissions at excitation wavelengths of 400 and 800 nm.** Waveforms of the **a** measured and **b** calculated THz emissions. **c** Calculated photocurrent distributions. The current intensity at depths below 0.8 µm is enlarged by a factor of 5. The blue and red solid lines represent the results for PN1 and SIM1 at the excitation wavelengths of 400 and 800 nm, respectively. The pink dash-dotted lines correspond to the results for PN3 and SIM3 under 800 nm excitation.

**Supplementary 8: Stability of the THz emission from the semiconductor surface.** To demonstrate the validity of the noise level determination method, we show the long-term stability of the THz emission from the semiconductor surface electric field in Supplementary Fig. S8. We fixed the time delay at the peak position of the THz waveform (black arrow in the inset: 10.24 ps) and monitored the peak intensity for more than 300 seconds. This plot shows no significant change in the THz peak amplitude, suggesting long-term stability. Then, we compared the noise levels with/without THz emission. Note here that we cut the excitation laser until 27 s, then started the laser illumination (the red arrow indicates the start of the excitation). This means that only background noise was detected before 27 s and the signal noise appeared after 27 s. Calculated standard deviations for before and after 27 s are 2.4×10^-3^ (calculated from the signal within 1.9 – 24.4 s) and 2.1×10^-3^ (calculated from the signal within 51.9 – 146.2 s), respectively. In addition, we mention the repeatability of the THz emission signals. Our previous report showed that no significant change appeared in the repeated measurements of the THz emission from a semiconductor surface, indicating the repeatability of the THz emission measurement (see Supplementary Note 3 in Ref. 3. These results showed the stability and repeatability in THz emission measurements and suggested that the background noise is dominant in the noise of measurement in the present work, which can originate from Johnson-Nyquist noise and shot noise generated at a PCA.^4,5^

.


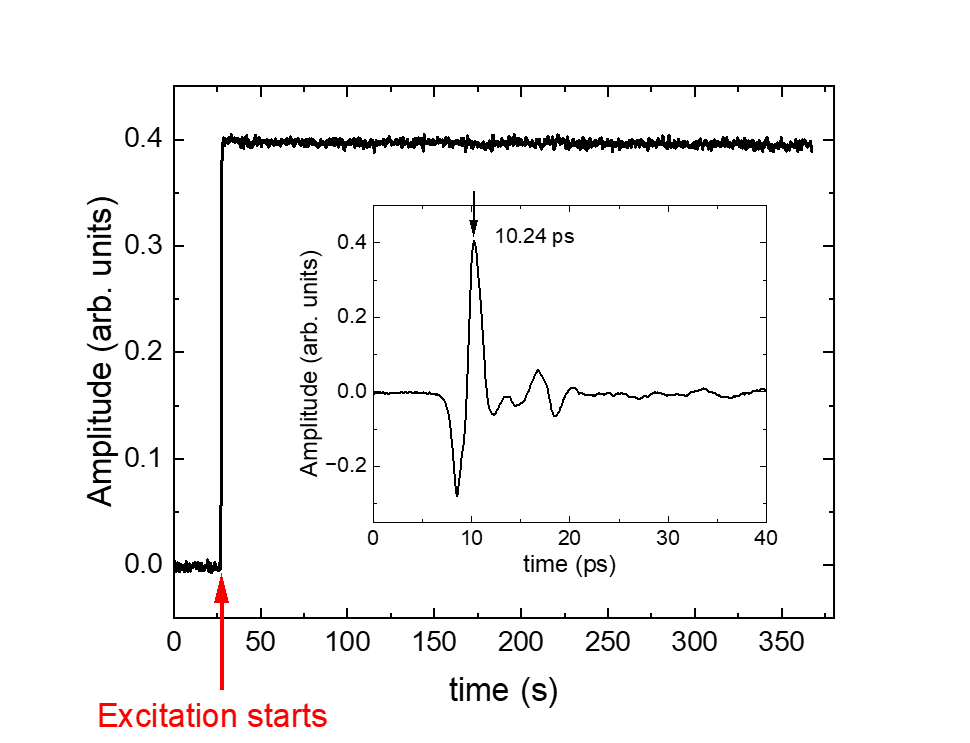


**Supplementary Fig. S8 | Long-term stability of the THz emission amplitude.** We plotted the peak amplitude of the THz emission, indicated by arrows in the insets, over a 300-second duration. The sample was excited after 27 s (indicated by the red arrow).

**Supplementary 9: Incident angle dependence of the THz emission amplitude.** The amplitude of the THz emission from a PN junction at a depth of *x* is expressed as Eq. (S14). Since mobility $\mu$ and diffusion potential $V_{D}$ vary with doping density, it is generally believed that extracting the doping density by another method, e.g., SIMS, is necessary to evaluate the PN junction depth for unknown samples. However, when a wafer is excited at an incident angle of $\theta_{\mathrm{in}}$ as shown in Fig. S9a, the THz emission amplitude $E_{\mathrm{THz}}(\theta_{\mathrm{in}})$ can be expressed as

| $E_{\mathrm{THz}}\left( \theta_{\mathrm{in}} \right)\propto\mu V_{D}I_{p0}\exp\left( -\frac{1}{\lambda_{\mathrm{eff}}\left( \theta_{\mathrm{in}} \right)}x \right)$ | (S16) |
| --- | --- |

where $\lambda_{\mathrm{eff}}(\theta_{\mathrm{in}})=\lambda_{L}\cos\theta_{\mathrm{in}}$ represents the effective penetration length in the depth direction. Consequently, normalized $E_{\mathrm{THz}}(\theta)$ with respect to the amplitude at $\theta=0$ can be expressed as

| $\mathrm{Norm}E_{\mathrm{THz}}\left( \theta_{\mathrm{in}} \right)=\frac{E_{\mathrm{THz}}(\theta_{\mathrm{in}})}{E_{\mathrm{THz}}(0)}=\exp\left( -\frac{1-\cos\theta_{\mathrm{in}}}{\lambda_{L}\cos\theta_{\mathrm{in}}}x \right)$ | (S17) |
| --- | --- |

As shown in this equation, the effect of variation in material parameters can be subtracted. This suggests that TES can probe the junction depth solely without any reference sample. The calculation of Eq. (S17) shows that the angle dependence of the THz emission varies with various junction depths, as shown in Supplementary Fig. S9b. For example, Norm$E_{\mathrm{THz}}$ = 0.5 is obtained at angles of *θ*_in_ = 72, 62, 56, and 50 deg for junction depths of *x* = 30, 60, 90, and 120 nm, respectively. We used an excitation wavelength of 400 nm (penetration depth of 100 nm) for the calculation. This result indicates that we can evaluate the PN junction depth inside unknown wafers by TES.


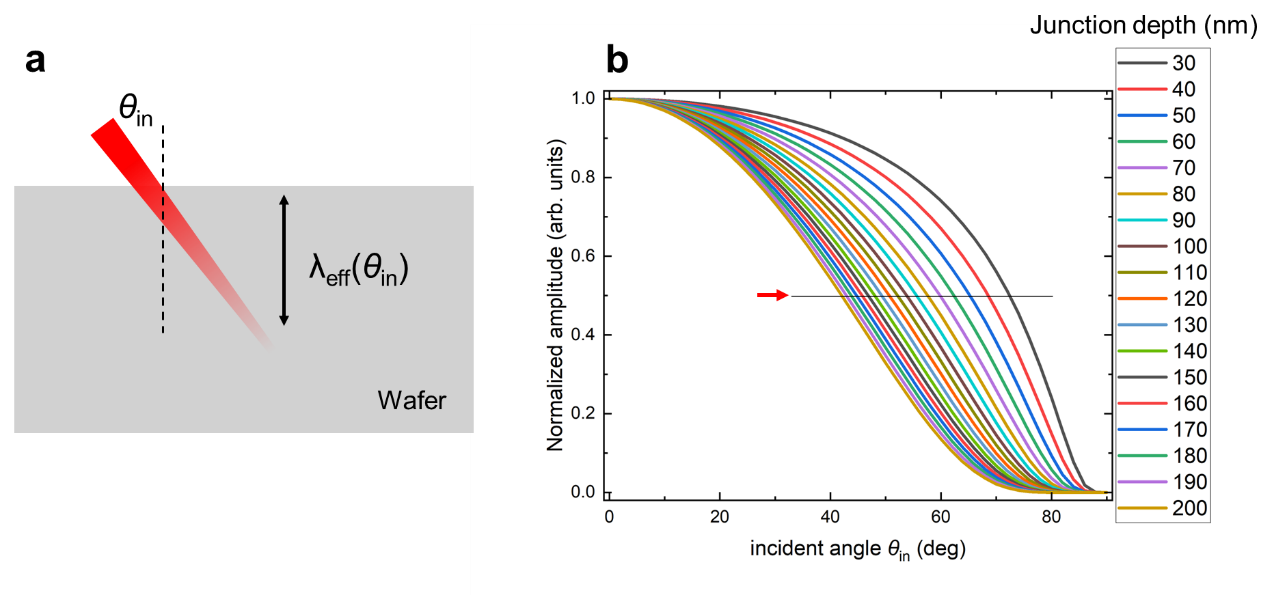


**Supplementary Fig. S9 | Incident angle dependence of the THz emission from PN junction.** **a** Illustration of the incident angle, *θ*_in_, and effective penetration depth, $\lambda_{\mathrm{eff}}(\theta_{\mathrm{in}})$. **b** Normalized THz emission amplitude vs incident angle with various junction depths calculated from Eq. (S17).

**Supplementary 10: Improvement in the depth sensitivity by 45-degree excitation/detection scheme.** In the present work, we employed the incident angle of 45 degrees to enhance the THz emission. At the PN junction, THz emissions are generated by the transient dipole source formed perpendicular to the sample surface due to the transient photocurrent, where the THz waves emitted to the air side (i.e., outside the semiconductor) are small owing to the radiation angle.^6^ In addition, the intensity of the THz generation from silicon is known to be weak.^7^ Therefore, to obtain a stronger emission, it is necessary to use the phased-array effect with a large excitation beam spot size.^8,9^ In the phased-array effect, it has been observed that the excitation with an incident angle of 30–60 degrees generates larger THz emissions than the excitation normal to the wafer surface.^8,9^ Therefore, we employed the 45-degree incident angle to enhance the signal-to-noise ratio in this work. It should be noted that the THz emission from transient carrier acceleration does not need the phase-matching condition.

In addition, we chose the 45-degree reflection configuration to improve the sensitivity to PN junction depths. We compared the two detection configurations: reflection and transmission configuration, as shown in Supplementary Figs. S10a and S10b, respectively, to understand the role of THz absorption in this technique. In the reflection setup, THz waves generated at the PN junction propagate through the n-Si layer and are detected at a 45-degree reflection angle. In the transmission setup, THz waves propagating through the p-Si and ud-Si layers are detected at a 45-degree transmission angle. We measured the THz waveforms emitted from PN1–PN3 by these two setups and plotted them in Supplementary Fig. S11. THz emission amplitude in Supplementary Fig. S11a strongly depended on the PN junction depth, while that in Supplementary Fig. S11b did not. This difference is attributed to the absorption of the THz waves by free carriers in n-Si or p-Si layers. For reflected THz emission, a deeper PN junction (or a thicker n-Si layer) results in larger absorption in the n-Si layer. Furthermore, a thicker n-Si layer makes a smaller THz emission at the PN junction due to the penetration of excitation pulses. The combination of them enhances the sensitivity to the PN junction depth. On the other hand, the absorption of transmitted THz waves in the p-Si layer has an opposite tendency; a deeper PN junction depth (which means a thinner p-Si layer) induces a smaller absorption. As a result, amplitude changes obtained by the reflection configuration were larger than those obtained by the transmission setup. This result suggests that the reflection configuration makes the technique sensitive to the PN junction depth.


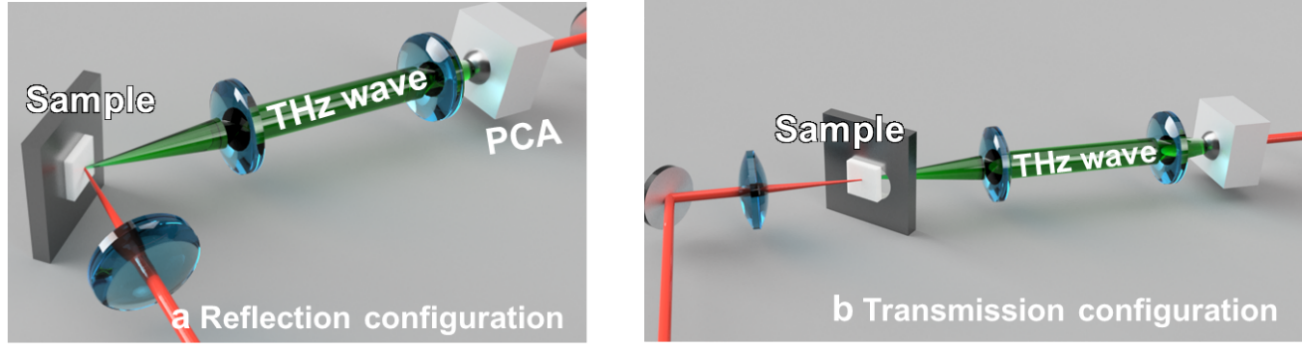


**Supplementary Fig. S10 | Two different configurations for the THz detection.** **a** a reflection setup and **b** a transmission setup.


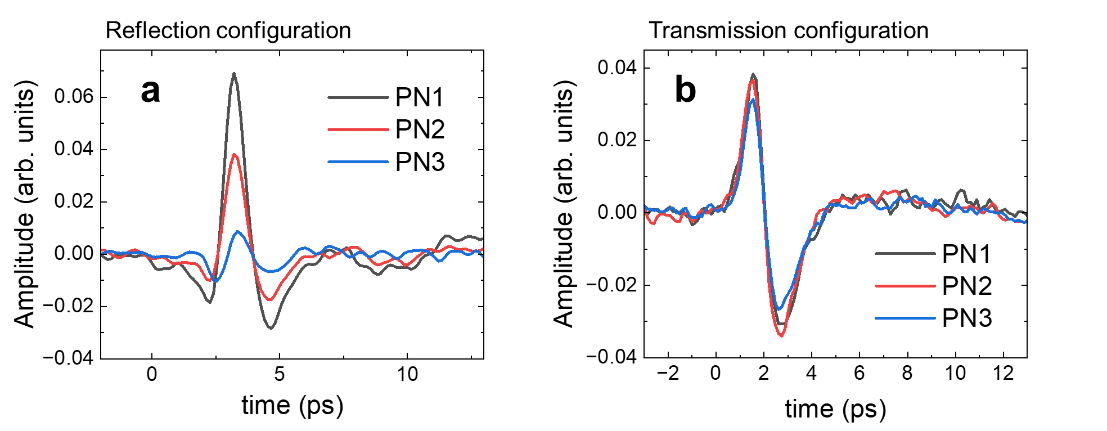


**Supplementary Fig. S11 |** THz emission waveforms obtained by **a** the reflection configuration and **b** the transmission configuration.

**References:**

1. Liu, K., et al. Terahertz radiation from InAs induced by carrier diffusion and drift. *Physical Review B* **73**, 155330 (2006).
2. Apostolopoulos, V. & Barnes, M. E. THz emitters based on the photo-Dember effect. *Journal of Physics D: Applied Physics* **47**, 374002 (2014).
3. Murakami, F. et al. Enhanced luminescence efficiency in Eu-doped GaN superlattice structures revealed by terahertz emission spectroscopy. *Communications Materials* **4**, 100 (2023).
4. Shi, W. et al. A High Performance Terahertz Photoconductive Antenna Array Detector With High Synthesis Efficiency. *Frontiers in Physics* **9**, 751128 (2021).
5. Wang, N. & Jarrahi, M. Noise analysis of photoconductive terahertz detectors. *Journal of Infrared, Millimeter, and Terahertz Waves* **34**, 519–528 (2013).
6. Johnston, M. B., et. al. Simulation of terahertz generation at semiconductor surfaces. *Physical Review B* **65**, 165301 (2002).
7. Hoyer, P., et. al. Terahertz emission from black silicon. *Applied Physics Letters* **93**, 091196 (2008).
8. Inoue, R., et. al. Angular dependence of terahertz emission from semiconductor surfaces photoexcited by femtosecond optical pulses. *Journal of the Optical Society of America B* **26**, A14–A22 (2009).
9. Mannan, A. et al. A better understanding of terahertz emission from semiconductor surfaces with a phased-array effect. *AIP Advances* **11**, 125021 (2021).
